# Omicron: A new face of COVID‐19 pandemic

**DOI:** 10.1002/hsr2.526

**Published:** 2022-02-18

**Authors:** Amjad Khan, Salma Bibi, Hafsa Kanwal, Umm‐e‐Kalsoom, Hidayat Hussain

**Affiliations:** ^1^ Department of Pharmacy Quaid‐i‐Azam University Islamabad Pakistan; ^2^ Department of Bioorganic Chemistry Leibniz Institute of Plant Biochemistry Halle (Saale) Germany

Omicron, the B.1.1.529 strain of Severe Acute Respiratory Syndrome Coronavirus 2 (SARS‐CoV‐2), has been reported to the World Health Organization (WHO) on November 24, 2021, by the National Institute of Communicable Diseases (NICD), South Africa. Whereas the first confirmed case of B.1.1.529 variant was reported by NICD (South Africa) in a sample collected on November 9, 2021. Virus Evolution Expert Working Group constituted by WHO in June 2020 to study the development, mutations, and variants of SARS‐CoV‐2 has classified Omicron as a Variant of Concern (VOC) on November 26, 2021.^1^

According to the Network for Genomics Surveillance in South Africa (NGS‐SA), the variant (B.1.1.529) possesses “quite uncommon constellations of mutations.” The mutations in B.1.1.529 may lead to certain changes in the pattern of disease, such as an increase in transmissibility, change in COVID‐19 etiology, increase in virulence, change in symptomatic presentation, efficacy of public health and social measures or available diagnostic tests, vaccines, and therapeutics; thus, implicating a global health concern.^1^ These amino acid mutations occur in the Receptor Binding Domain (RBD) of virus. Mutations H655Y, N679K, and P681H encoding the spike protein are significant for the virus's entry into human cells thus enhancing transmissibility; D614G, N501Y, and K417N make omicron more infectious. Omicron also has several mutations (eg, R203K and G204R) that were previously found in other variants; Alpha, Gamma, and Lambda.^2^ Omicron has been evolved into three lineages; BA.1, BA.2, and BA.3, most popular are BA.1.^3^ Features of Omicron are summarized in Table 1.

**TABLE 1:** Main features of Omicron

Mutations	Omicron has more than 50 mutations, 26‐32 of which are on spike protein; critical mutations are, S371L, G339D, S375F, S477N, S373P, T478K, N501Y, K417N, Q496S, Q493R, Q498R, Y505H, H655Y, N679K, D614G, and P681H.^2^
Symptoms	Runny nose, fatigue (mild–severe), headache, sore throat, and sneezing. Omicron is more likely to infect upper respiratory tract than lungs, thus making it more infectious and less deadly than previous variants.^4^, ^5^
Vaccines efficacy	Populations having no immunization status are at higher risk of infections from Omicron with more severe attack.^2^ According to UK Health Security Agency (UKSHA), third COVID‐19 vaccine dose (booster) can provide higher protection against Omicron and reduces the risk of hospitalization up to 88% in infected patients.^6^
Reinfection rate	In comparison to other VOCs, Omicron poses a higher risk of reinfection.^1^ A person who was previously infected with COVID‐19, has 5^1/2^% more risk of reinfection with Omicron than reinfection with Delta variant.^7^

Polymerase Chain Reaction (PCR) assay, being used for the detection of previous strains, continues to detect the Omicron variant.^1^ Corticosteroids and IL‐6 receptor blockers have shown efficacy to manage severe infections with Omicron as for its antecedents.^2^ Food and Drug Administration has recently approved Paxlovid and monoclonal antibodies (Softrovimab) that have shown promising results to fight against Coronavirus; Molnupiravir is also under consideration.^8^ A new Coronavirus antibody, Xevudy (Sotrovimab), has been approved by the Medicines and Healthcare Products Regulatory Agency and is claimed to have effectiveness against Omicron.^9^ The detection of Omicron has triggered the global alarm, WHO reports that the spread of omicron is so fast that has not been observed for any previous Coronavirus strains.^1^ So far, Omicron has been detected in more than 108 countries across all continents.^6^ Emergence of Omicron is a stark reminder of ongoing threat by continuous evolution of COVID‐19 pandemic. There is a critical need for high‐level global immunization coverage and changes in vaccination policies. WHO has released the Strategy to Achieve Global COVID‐19 Vaccination by Mid‐2022, that is, 70% total population coverage by Mid‐2022. Modest decrease in vaccine protection has been reported over 6 months after the primary vaccination and the administration of booster dose has shown improvement in protection level. Regulatory and advisory bodies recommend the administration of booster dose 6 months after the primary vaccination and have assessed a favorable risk‐benefit ratio.^1^

After reporting of first case in Karachi on December 13, 2021, Pakistan grapples with the Omicron‐driven fifth COVID‐19 wave. National Command and Operations Center (NCOC) Pakistan has announced to adopt a “zero tolerance” policy to counter the new strain of SARS‐CoV‐2. National vaccination strategy has been annunciated by NCOC, that is, to start a mass vaccination campaign from December 1, 2021. All provinces and concerned authorities have been urged to show a “zero tolerance” policy regarding obligatory vaccination regime. The national vaccine strategy will focus those individuals on priority who have not been vaccinated at all and will administer booster dose to already vaccinated people.^10^

Three categories of people; immunocompromised, healthcare workers, and citizens above 30 years of age, will be entertained for booster dose in first phase, those receiving booster dose must have received their last vaccine dose six months ago. The vaccination teams have been deployed at various public spots along with the establishment of call centers to track people who have not completed their two‐dose primary vaccination schedule. Travel restrictions have been imposed on people coming from high‐risk countries along with necessary measures on airports to check the vaccination status and testing of expatriates. In addition, sampling rate has also been increased, and the contact tracing system is revived with emphasis to rejuvenate it with more resources and increased efficiency. The target to inoculate 70 million people with vaccination has been achieved by the end of 2021. The vaccination teams are working with full zeal to comply with the NCOC directions and WHO's vaccination targets.^10^, ^11^

Studies on the assessment of transmissibility, severity of infection, diagnostic tests, performance of vaccines, and effectiveness of treatment for the new strain (Omicron) are still underway. The emergence of the newest strain demonstrates that the pandemic is far from ending, and appropriate behavior is crucial for breaking the transmission chain that is masking, social distancing, good ventilation in all common locations, and regularly washing or sanitizing hands and surfaces, meanwhile, mass vaccination campaigns to achieve global vaccination coverage target are imperative.

## FUNDING

None declared.

## AUTHOR CONTRIBUTIONS

Conceptualization: Amjad Khan.

Writing – original draft: Amjad Khan, Salma Bibi, Hafsa Kanwal, Umm‐e‐Kalsoom.

Writing – review and editing: Amjad Khan, Salma Bibi, Hidayat Hussain.

All authors read and have approved the final manuscript.

Amjad Khan was responsible for the concept and literature search; Salma Bibi, Hafsa Kanwal, and Umm‐e‐Kalsoom drafted the letter; Amjad Khan and Hidayat Hussain revised the letter. Hafsa Kanwal and Umm‐e‐Kalsoom helped in data collection; Amjad Khan, Hidayat Hussain, and Salma Bibi helped in data analysis and interpretation.

## CONFLICT OF INTERESTS

The authors have no conflict of interest to declare.
